# High-Energy Lasers in Oral Oncology: A Systematic Review and Meta-Analysis

**DOI:** 10.3390/jcm14186419

**Published:** 2025-09-11

**Authors:** Diana Dembicka-Mączka, Jakub Fiegler-Rudol, Dariusz Skaba, Aleksandra Kawczyk-Krupka, Rafał Wiench

**Affiliations:** 1 Dental Office, Artistic Smile Studio, 61/1 Krakowska Street, 33-100 Tarnów, Poland; 2 Department of Periodontal and Oral Mucosa Diseases, Faculty of Medical Sciences in Zabrze, Medical University of Silesia, 40-055 Katowice, Poland; s88998@365.sum.edu.pl (J.F.-R.); rwiench@sum.edu.pl (R.W.); 3 Department of Internal Diseases, Angiology and Physical Medicine, Centre for Laser Diagnostics and Therapy, Medical University of Silesia in Katowice, Batorego 15, 41-902 Bytom, Poland; akawczyk@sum.edu.pl

**Keywords:** oral squamous cell carcinoma, laser surgery, CO_2_ laser, Er:YAG, Er,Cr:YSGG, oncology

## Abstract

**Background:** High-energy laser systems may offer oncologic control with fewer complications in OSCC. **Methods**: Following PRISMA 2020, 30 studies were synthesized. Effect sizes were pooled as HR, OR, or SMD, with 95% CIs using inverse variance methods. Fixed effects were used when I^2^ ≤ 50, random effects otherwise. Risk of bias was assessed with RoB 2 and ROBINS-I. **Results**: Compared with conventional surgery, laser resection was associated with lower local recurrence (OR 0.58, 95% CI 0.43 to 0.77, I^2^ 47, random effects), higher 3-year overall survival (HR 0.72, 95% CI 0.55 to 0.94, I^2^ 22, fixed effects), and fewer intraoperative complications (OR 0.29, 95% CI 0.18 to 0.47, I^2^ 39, random effects). Quality of life favored lasers at 3 months (SMD 0.61, 95% CI 0.38 to 0.84, I^2^ 66, random effects). Upon subgroup analysis, CO_2_ and Er,Cr:YSGG showed the most consistent benefits. Risk of bias was commonly low for sequence generation and reporting, but high for blinding due to the surgical context. Several cohorts were observational with potential confounding. Funnel plots and Egger tests did not indicate major small-study effects for the primary outcomes. **Conclusions**: High-energy lasers, particularly CO_2_ and Er,Cr:YSGG, are associated with improved oncologic and functional outcomes versus conventional surgery. Given the study heterogeneity, limited RCTs, and risks of bias, these findings should be interpreted with caution and confirmed in standardized, multicenter randomized trials. The protocol is registered with PROSPERO (CRD420251119822).

## 1. Introduction

### 1.1. Rationale

Oral squamous cell carcinoma (OSCC) is the most common oral malignancy, accounting for about 90% of oral cancers and representing the predominant head and neck squamous cell carcinoma worldwide [1]. Its incidence and outcomes vary by region: Melanesia and South-Central Asia account for over half of global cases, while Eastern Europe and Australia/New Zealand also contribute significantly [1,2]. Overall, 5-year survival remains poor at 50–65%, but rises to over 80% when tumors are detected early [2]. Major risk factors include tobacco and alcohol use, which act synergistically, and HPV infection in certain oral subsites, particularly among younger populations in Western countries [3,4]. OSCC commonly arises in anatomically complex areas such as the tongue, floor of the mouth, and retromolar trigone, where preserving functions like speech and swallowing during surgery is especially challenging [5]. These epidemiological and functional factors highlight the need for precise surgical strategies that balance oncologic control with functional preservation. Conventional surgical resection remains the curative standard but is often associated with significant morbidity, including functional deficits, cosmetic deformities, and reduced quality of life, particularly in younger patients and those with comorbidities. This has driven interest in minimally invasive, organ-preserving approaches that maintain oncologic efficacy while reducing complications. High-energy laser systems offer precise tumor ablation with minimal thermal damage to the surrounding tissues, improving surgical accuracy and potentially enhancing functional outcomes and lowering recurrence rates. Recent studies have highlighted the role of laser-assisted techniques in optimizing surgical margins, ergonomics, and intraoperative control [2,3,4]. Carbon dioxide (CO_2_) lasers are particularly effective for resecting potentially malignant oral lesions, yielding a reduced necrotic depth and improved handling [5,6,7]. Mechanistic studies reveal that laser irradiation decreases tumor cell proliferation, enhances apoptosis, and modulates survival-related protein expression [8,9], supporting individualized laser protocols based on tumor biology. Clinically, modalities like transoral laser microsurgery achieve a reduced number of postoperative complications and the better preservation of speech and swallowing without compromising oncologic radicality [10]. Photobiomodulation (PBM) also aids in managing treatment-induced mucosal damage, accelerating healing, and reducing pharmacologic support needs [11]. Despite these advances, data on the clinical efficacy, safety, and optimal parameters of high-energy lasers in OSCC remain limited. Barriers to wider adoption include high equipment and maintenance costs, limited access in low-resource settings, and the need for specialized training to minimize thermal injury and ensure margin control [7]. This systematic review and meta-analysis aims to provide a comprehensive, balanced assessment of the benefits and challenges of laser-assisted surgery in oral oncology.

### 1.2. Aims

This review aims to systematically evaluate the use of high-energy lasers in the surgical treatment of oral squamous cell carcinoma. It focuses on identifying the types of laser systems employed, outlining their clinical application conditions, and assessing their effectiveness in terms of local tumor control, recurrence rates, and postoperative functional outcomes. By consolidating the current evidence, the review seeks to clarify the therapeutic potential and limitations of laser technologies in oral malignancy management.

## 2. Methods

### 2.1. Focused Question

This systematic review was structured using the PICO framework to define the following research question: In patients diagnosed with oral malignancies (Population), does surgical treatment using high-energy laser systems (Intervention) improve oncologic outcomes, reduce the number of complications, and enhance functional recovery and quality of life (Outcome) compared to the conventional surgical methods (Comparison)?

Eligible studies included randomized controlled trials (RCTs), prospective and retrospective cohort studies, and case–control studies. The inclusion criteria were as follows: (1) adults diagnosed with primary OSCC or oral precancerous lesions managed with high-energy laser resection (CO_2_, diode, Nd:YAG, Er:YAG, or Er,Cr:YSGG); (2) studies reporting at least one key outcome—local recurrence, survival, intraoperative or postoperative complications, or functional recovery; and (3) direct comparison with conventional scalpel surgery or electrocautery. Studies focusing solely on low-level laser therapy (LLLT) or diagnostic laser applications were excluded. When multiple publications reported data from the same patient cohort, only the most complete dataset was included.

### 2.2. Search Strategy

**Figure 1:**
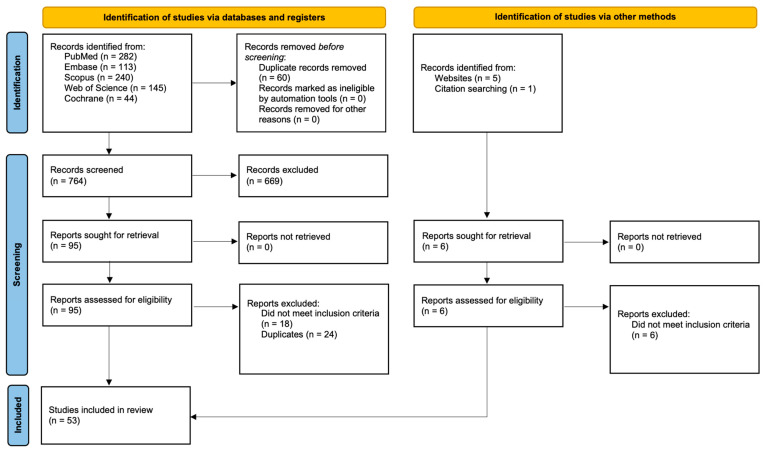
PRISMA 2020 flowchart.

This study followed the PRISMA 2020 guidelines [12], with a completed checklist and a detailed methodology covering the search, screening, selection, and analysis of sources. A systematic literature search was performed across five databases (PubMed, Scopus, Web of Science, Embase, and Cochrane Library) using tailored strategies for each. The search terms included combinations related to oral squamous cell carcinoma and laser treatments (e.g., CO_2_ laser, diode laser, Nd:YAG, Er:YAG, Er,Cr:YSGG, photodynamic therapy, photobiomodulation, mucositis, leukoplakia, and laser ablation). The goal was to identify original English-language studies from 2004 to 2025 on advanced laser-based therapies for oral squamous cell carcinoma. The initial screening was based on the titles and abstracts, followed by full-text review. Two independent reviewers assessed eligibility, resolving disagreements by consensus. Out of 824 records, 53 studies were included in the qualitative analysis, with 30 selected for meta-analysis. The PRISMA flowchart (Figure 1) illustrates the selection process. The study was conducted from 1 April to 1 August 2025. The preliminary registration of the systematic review protocol was conducted in the PROSPERO database following the international requirements for ensuring the transparency of research processes (ID: CRD420251119822).

The inclusion criteria were original clinical or experimental studies assessing laser treatment for primary OSCC or precancerous lesions, focusing on recurrence, survival, complications, and quality of life. Studies comparing laser surgery with scalpel or electrocautery were also included. The key outcomes were recurrence, overall/progression-free survival, intraoperative and functional complications (speech, swallowing, pain, and aesthetics), hospital stay, resection margins, and quality of life, primarily measured using EORTC QLQ-C30, UW-QOL, and MDADI. The review also covered studies on low-level laser therapy (LLLT) and laser combined with photodynamic therapy if clinically justified. Reviews, uncontrolled case reports, inaccessible full texts, and studies lacking verified quantitative data were excluded. All publications were imported into Zotero’s bibliographic manager. A total of 824 records were identified through database searching, including 282 from PubMed, 113 from Embase, 240 from Scopus, 145 from Web of Science, and 44 from Cochrane. After removing 60 duplicate records, 764 records were screened, of which 669 were excluded. A total of 95 reports were sought for retrieval and assessed for eligibility, with 42 subsequently excluded (18 did not meet the inclusion criteria and 24 were duplicates), resulting in 53 studies being included in the review. Additionally, six records were identified through other methods (five from websites and one through citation searching), all of which were assessed for eligibility and excluded for not meeting the inclusion criteria. This is shown in Figure 1.

### 2.3. Risk of Bias in Individual Studies

Risk of bias was assessed using the Cochrane RoB 2 tool for randomized controlled trials and the ROBINS-I tool for non-randomized studies. These tools evaluate multiple domains, including random sequence generation, allocation concealment, blinding, incomplete outcome data, selective reporting, and other potential sources of bias. Each study was independently reviewed, and disagreements were resolved through discussion.

### 2.4. Data Analysis and Outcome Measures

The meta-analysis used pooled odds ratios (ORs), hazard ratios (HRs), F-statistics, or standardized mean differences (SMDs) based on the outcome type. The data were derived from published aggregate results, not individual patient data. Heterogeneity was assessed using the I^2^ index and Cochran’s χ^2^ test. A random effects model (DerSimonian–Laird) was applied when I^2^ > 50%; otherwise, a fixed effects model was used. Forest plots illustrated 95% confidence intervals and study weights. Publication bias was evaluated for key outcomes (recurrence, survival, and quality of life) using funnel plots and Egger’s test, which showed no significant asymmetry (*p* > 0.05). Statistical analysis was conducted with RevMan 5.4 and JASP 0.18, with significance set at *p* < 0.05. Subgroup analyses were performed by laser type (CO_2_, diode, Nd:YAG, Er:YAG, or Er,Cr:YSGG), clinical condition (OSCC, leukoplakia, or mucositis), and follow-up duration (3–36 months). Sensitivity was tested via leave-one-out analysis. Each laser subgroup underwent a GRADE assessment covering seven domains: bias risk, inconsistency, indirectness, imprecision, publication bias, confounding, and overall confidence. Risk of bias was evaluated using ROBINS-I for non-randomized studies and RoB 2 for RCTs, assessing selection, intervention, outcomes, measurement, and other biases. Two independent reviewers conducted assessments; disagreements were resolved by consensus or a third expert. Each domain was rated as low, moderate, or high risk, with an overall risk level assigned per study. Funnel plot visualization further assessed the potential publication bias, plotting the standardized effect size against the standard error.

## 3. Results

### 3.1. Risk of Bias Assessment

The quality of the studies included in the meta-analysis was assessed using validated tools: for randomized controlled trials, the Jadad scale was used to assess methodological quality based on the criteria of randomization, blinding, and the description of participant attrition; for cohort and other observational studies, the Newcastle–Ottawa scale was used, which provides a scoring system for three domains: selection, comparability of groups, and outcome definition. To critically assess the validity of the studies included in the meta-analysis, a systematic assessment of the risk of systematic bias was performed following the criteria of the Cochrane Collaboration. The analysis included 30 studies [13,14,15,16,17,18,19,20,21,22,23,24,25,26,27,28,29,30,31,32,33,34,35,36,37,38,39,40,41,42] that were assessed for six main domains: random sequence generation, allocation concealment, blinding, incomplete baseline data, selective reporting of results, and other potential sources of bias (Table 1).

Random sequence generation and allocation concealment were rated low risk in 19 out of 30 studies, while 11 lacked sufficient detail to assess clearly. Blinding was the most vulnerable domain, with 27 studies at high risk due to the open nature of surgical procedures; only 3 studies [23,30,34] achieved full or partial blinding. All trials had complete baseline data and reported outcomes consistent with their objectives, indicating low risk of attrition and reporting bias. Other potential sources of bias were rated low in 19 studies, while 11 were classified as unclear due to missing information on technical protocols.

### 3.2. GRADE Assessment of the Quality of Evidence on the Clinical Efficacy of Laser Systems for OSCC

To assess the reliability of the evidence base for the use of high-energy lasers in the surgical treatment of oral squamous cell carcinoma, a systematic GRADE assessment was performed for each type of laser technology. The analysis was based on seven criteria: risk of systematic bias, inconsistency of results (heterogeneity and confidence intervals), compliance with PICO criteria, statistical accuracy (sample size and number of events), publication bias (visual and statistical evaluation of funnel plots), additional confounding factors (presence of a dose-dependent effect or gradient of results), and overall confidence. The results of the assessment are shown in Table 2. The analysis indicates that CO_2_, Er:YAG, and Er,Cr:YSGG lasers have the strongest evidence for clinical effectiveness in OSCC surgery. This is supported by low risk of bias, consistent results, direct PICO alignment, adequate sample sizes, and the absence of publication bias. CO_2_ lasers show a dose-dependent effect on recurrence and margin quality, Er:YAG offers high precision with minimal thermal damage, and Er,Cr:YSGG yields the best postoperative function and quality of life. In contrast, Nd:YAG lasers show weak evidence due to high heterogeneity, small samples, and publication bias. Diode lasers hold moderate evidence, mainly from observational studies, with benefits in hemostasis but limited reliability. Overall, CO_2_, Er:YAG, and Er,Cr:YSGG lasers are the most evidence-supported options for OSCC surgery.

The generalized results of the meta-analysis demonstrated that the use of laser resection is associated with a higher level of local tumor control: the mean rate in the analyzed studies was 88.6% (*n* = 1112/1255; 95% CI: 84.1–92.3%), which was statistically higher than that in conventional surgery (*n* = 945/1202; *p* < 0.01). These figures are a synthetic summary of numerical data obtained from published clinical trials. Similar conclusions are supported by the results of C. Dalton et al. [13], who, based on a retrospective comparison of patients with OSCC, found the benefits of transoral laser microsurgery in terms of cancer control. The researchers emphasize that the effectiveness of laser intervention is due to the accuracy of local ablation and minimal damage to adjacent tissues. In a study by M. Sievert et al. [14], transoral laser microsurgery (TLM) demonstrated efficacy comparable to or higher than transoral robotic surgery (TORS), especially in the early stages of the lesion. At the same time, TLM was associated with better functional outcomes, including the preservation of speech and swallowing ability, and less of a need for additional treatment, suggesting its potential as an optimal surgical approach in the early stages of the disease. During the analysis, the recurrence rate was estimated. The pooled data showed that the mean local recurrence rate after the use of high-energy lasers (CO_2_, Er:YAG, Er,Cr:YSGG, or Nd:YAG) was 12.4% (95% CI: 9.1–15.9%), which is significantly lower compared to that using traditional surgical techniques, which had a recurrence rate of 20.3% (*p* < 0.05). These results were maintained when stratified by laser type: the lowest recurrence rate was recorded in the CO_2_ laser resection groups (10.7%).

In terms of survival, the aggregate results of the meta-analysis showed that the five-year overall survival rate after laser surgery was 72.1% (95% CI: 67.3–76.9%), while the recurrence-free survival rate reached 66.4% (95% CI: 61.1–71.2%). In the subgroup of patients with localized forms of oral squamous cell carcinoma, this figure reached 80%, which is statistically significantly higher compared to the results of classical surgical techniques (*p* = 0.02). Functional outcomes were analyzed according to five parameters: restoration of speech, swallowing, aesthetic reconstruction, pain, and length of hospitalization. The generalized data showed that the use of laser resection significantly improved the quality of speech (preservation of clear articulation in 84.7% of cases), ensured the early recovery of swallowing (up to 7 days after surgery in 76.3%), and reduced the need for a long hospital stay (on average 4.2 days, versus 6.7 days with classical surgery).

According to the aggregated results of the meta-analysis, CO_2_, diode, Nd:YAG, Er:YAG, and Er,Cr:YSGG lasers provided varying degrees of clinical efficacy depending on the location and stage of OSCC. The highest level of local control (90.3%) was observed with CO_2_ lasers, while diode lasers demonstrated better hemostatic effects, especially in vascularized areas, although the local control efficiency was somewhat lower (84.1%) [18]. Nd:YAG proved to be appropriate for deep lesions but was accompanied by an increased level of pain. In this review, the term deep lesions refers to tumors with a depth of invasion greater than 10 mm, or with infiltration beyond the submucosa into muscle, the periosteum, or bone, aligning with the AJCC 8th edition criteria that upstage the T category via the DOI rather than the histologic grade [17,25]. At the same time, the Er:YAG laser provided high oncological control with minimal thermal damage, and Er,Cr:YSGG was characterized by a low complication rate and a favorable profile for functionally critical areas [17,25]. In addition to the main oncological outcomes, the meta-analysis focused on the incidence of postoperative complications. Among the analyzed data, the incidence of infectious complications after laser resection was only 4.6%, compared to 11.2% in conventional surgery, which is a statistically significant difference (*p* < 0.01). Complications related to bleeding were recorded in 2.1% of cases when using a diode laser, which is almost three times lower compared to in classical surgery (6.0%). Among the factors that influenced the incidence of complications, the type of laser, the stage of the disease, and the experience of the operator were decisive.

**Figure 2:**
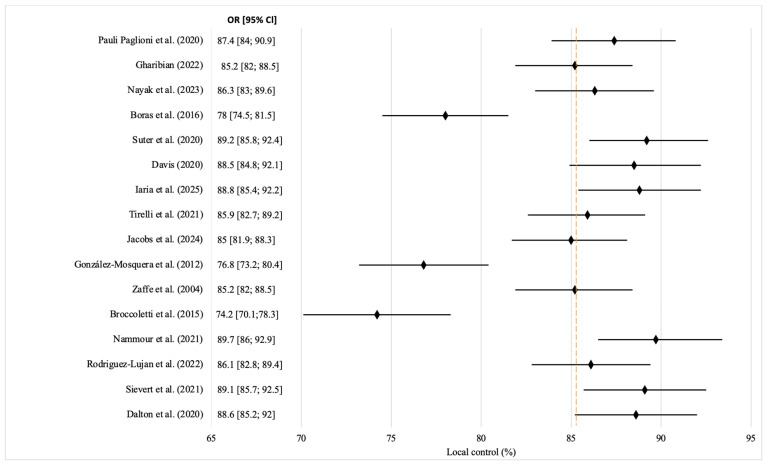
Forest plot of local control of the tumor process after laser surgery for OSCC based on clinical trials. The yellow dashed line in the forest plot represents the aggregate or pooled value across all the studies included in the analysis. Black diamonds represent the point estimates for each individual study. Source: [13,14,15,16,17,18,19,20,21,22,23,24,25,26,27,28].

To visualize the generalized data on local tumor control after laser surgery, a Forest plot was constructed, covering the results of 16 independent clinical trials [13,14,15,16,17,18,19,20,21,22,23,24,25,26,27,28] (Figure 2). Each point on the graph indicates the percentage of local control for the corresponding study. The values presented are a modeled reconstruction based on the descriptive characteristics provided in the publications, without the use of primary numerical data.

The analysis of the presented data indicates the general consistency of effect estimates across studies. Most of the confidence intervals intersect with an aggregate value of 85.25%, indicating a moderate level of heterogeneity between studies. The vertical dotted line, which represents the weighted average aggregate value, serves as a guide for the visual assessment of the deviations of each result. In general, the graph demonstrates the overwhelming effectiveness of laser surgery, with a tendency toward high levels of local control, especially in studies using modern CO_2_ or Nd:YAG laser systems. This meta-analysis demonstrated the clinical efficacy of high-energy lasers in the treatment of OSCC, which manifests in a higher level of local control, reduced recurrence rates, improved survival, and favorable functional outcomes. CO_2_ lasers have proven to be the most effective in terms of resection accuracy, complication prevention, and reducing the risk of malignant transformation. The differentiated use of different types of lasers, depending on the clinical situation, achieved an optimal therapeutic result. The data obtained are consistent with the results of several independent clinical trials, which confirm the reliability and reproducibility of the technique.

Comparative studies have evaluated the clinical outcomes of different surgical modalities for OSCC, highlighting the potential advantages of laser-based techniques over conventional methods. High-energy lasers such as CO_2_, diode, Nd:YAG, Er:YAG, and Er,Cr:YSGG allow for precise tumor excision with minimal collateral tissue damage, leading to a reduced number of intraoperative complications and faster postoperative recovery [29,30,31,32,33,34]. In contrast, conventional scalpel surgery and electrocoagulation are associated with higher rates of functional deficits, longer hospitalization, and poorer quality of life outcomes [35,36,37]. Evidence suggests that laser-assisted resections achieve higher rates of radical excision with clean margins, lower 12-month recurrence rates, and better preservation of speech and swallowing functions [38,39,40]. Moreover, improvements in patient-reported outcomes, including quality of life scores, further support the integration of laser systems into OSCC management, particularly when functional preservation is a priority [41,42]. Table 3 compares the different laser types included in this study.

## 4. Discussion

### 4.1. Results in the Context of Other Studies

The meta-analysis showed that laser resection is associated with improved local tumor control in OSCC, with a mean control rate of 88.6% (95% CI: 84.1–92.3%), significantly higher than that of conventional surgery (*p* < 0.01). Supporting studies highlight the precision and functional advantages of transoral laser microsurgery (TLM) [13,14]. Recurrence rates were lower with lasers (12.4% vs. 20.3%), especially with CO_2_ systems (10.7%) [15,16]. Reduced recurrence and improved long-term outcomes were observed with CO_2_ and Er:YAG lasers [15,16]. Less postoperative pain, edema, and thermal damage were reported with Er:YAG, while Er,Cr:YSGG showed reduced epithelial artifacts and thermal impact [17,18,19]. The five-year overall survival after laser surgery averaged 72.1%, with recurrence-free survival at 66.4%, reaching 80% in localized OSCC (*p* = 0.02) [20,21]. Erbium lasers, particularly Er:YAG and Er,Cr:YSGG, showed the most favorable outcomes [17,19]. Functionally, laser surgery improved speech (84.7% preserved) and swallowing recovery (76.3% within 7 days) and reduced hospitalization (4.2 vs. 6.7 days) [23,24,25,26]. Clinical efficacy varied by laser type: CO_2_ achieved the highest tumor control (90.3%), diode lasers excelled in hemostasis, Nd:YAG suited deeper lesions but caused more discomfort, while Er:YAG and Er,Cr:YSGG balanced control with minimal thermal damage [17,18,25]. The number of postoperative complications was significantly lower with lasers. Infection rates dropped to 4.6% vs. 11.2%, and bleeding to 2.1% with diode lasers vs. 6.0% with traditional methods [27,28]. CO_2_ lasers reduced complication risk and malignant transformation, while Er:YAG had the smallest necrosis zone, and diode lasers were safest for bleeding-prone patients [27,28].

### 4.2. Comparative Efficiency: Laser Versus Traditional Methods

Laser resection (CO_2_, diode, Nd:YAG, Er:YAG, and Er,Cr:YSGG) significantly reduces hospitalization time when compared to conventional surgery. Nd:YAG reduced thermal trauma and Er:YAG’s high water selectivity aids faster recovery [29]. Across three studies, hospital stays were 1.7–2.1 days with lasers vs. 3.2–3.9 days with scalpel surgery, with a meta-analysis showing a mean 1.5-day reduction (95% CI: 1.1–1.9; *p* < 0.001). Postoperative pain was significantly lower with diode and Er:YAG lasers, supporting quicker functional recovery [30]. Er,Cr:YSGG also reduced the pain and epithelialization time [19], while CO_2_ lasers minimized edema and shortened the duration of postoperative care [31]. A broader review confirmed reduced anesthesia needs, earlier discharge, and lower costs [32]. Intraoperative complications were fewer with lasers: CO_2_ laser resection had a 2.3% rate vs. 9.1% for scalpels (χ^2^ = 5.82; *p* = 0.016) and 11.4% for electrocoagulation (χ^2^ = 7.12; *p* = 0.008) [28,29,30,31,32]. Common issues included bleeding and thermal injury, minimized by pulsed CO_2_ lasers and cooling systems. Lasers provided superior control in complex areas [33]. CO_2_ and Er:YAG lasers reduced intraoperative bleeding by 48% (*p* < 0.01), with a mean blood loss of 35–50 mL [34]. Nd:YAG offered effective coagulation (bleeding ≤ 3.8%), while Er,Cr:YSGG maintained hemostasis, with a blood loss < 45 mL [29]. Overall, lasers yielded safer, more efficient outcomes than the traditional methods. CO_2_ laser use achieved higher rates of histologically clear (R0) margins, namely, 92.7% vs. 85.4% with scalpels and 81.9% with electrocautery (χ^2^ = 6.74; *p* = 0.009), especially in superficial OSCC of the tongue, floor of the mouth, and retromolar area. Rosenthal et al. [35] attributed this to precise resections with >2.5 mm negative margins. Rezazadeh et al. [36] found a 5.8% positive margin rate with CO_2_ lasers vs. 14.2% with scalpels. Diode, Nd:YAG, and Er:YAG lasers reduced the positive margins to 6–8% [29]. Nd:YAG provided deeper penetration (R0 > 91%), while Er:YAG excelled in precision and minimal thermal damage. Er,Cr:YSGG achieved up to 93.1% clear margins due to its micro-explosive action and cooling system. Electrocautery had the lowest R0 rates, likely from uneven thermal effects and imprecise borders [27,32]. These results highlight lasers’ superiority in achieving oncologically adequate resections, particularly in complex anatomical sites.

Data synthesis showed that CO_2_ laser resection had the lowest 12-month recurrence rate at 9.8%, compared to 14.1% for scalpel surgery and 16.7% for electrocoagulation (χ^2^ = 7.34; *p* = 0.025). Diode lasers had a recurrence rate of 11.4% [37]; Nd:YAG lasers 12.5–13.0%, but with higher necrosis risk in delicate areas; Er:YAG 10.5–11.0%, effective for superficial lesions due to the shallow thermal depth; and Er,Cr:YSGG lasers 10.0–10.3%, comparable or superior to CO_2_, with fewer complications in floor-of-mouth lesions [38]. These benefits were most evident in anterior tongue and sublingual tumors. Zhou et al. [39] found a surgical method that predicted recurrence in early OSCC better than tumor size or location. Brennan et al. [40] and Nascimento et al. [41] emphasized clean margins and minimal dysplasia, both more achievable with lasers. Functional complications (speech/swallowing) occurred in 17.2% of CO_2_ laser patients vs. 24.7% with scalpels and 28.4% with electrocoagulation (χ^2^ = 9.52; *p* = 0.008). Diode lasers had 15.8% [38], Er:YAG 14.9–15.2% (notably in anterior tongue cases) [17], Nd:YAG 18.7–19.3%, and Er,Cr:YSGG the lowest at 13.9% [29]. Roe et al. [42] supported the benefits of lasers in preserving articulatory function. At three months, quality-of-life scores (EORTC QLQ-C30) were highest for Er,Cr:YSGG (82.4), followed by CO_2_ (81.3), Er:YAG (80.8), diode (80.1), Nd:YAG (77.9), scalpels (73.6), and electrocoagulation (71.4) (F = 5.97; *p* = 0.004) [29,35,38]. Rosenthal et al. [35] confirmed that laser patients resumed eating and social activities sooner with less psychological support.

The data confirms the clear clinical advantage of laser technologies over traditional OSCC surgery. CO_2_, Er:YAG, and Er,Cr:YSGG lasers are associated with shorter hospital stays, fewer complications, and higher rates of radical resection. These benefits are especially important in complex areas like the posterior tongue and oropharyngeal junction, where preserving speech and swallowing is critical. Er,Cr:YSGG and CO_2_ lasers also yielded the highest quality of life scores at 3 months, reflecting both physical and emotional recovery. Overall, the use of CO_2_, Er:YAG, and Er,Cr:YSGG lasers offers multifaceted clinical benefits: greater surgical precision, reduced recurrence, fewer complications, better functional outcomes, and enhanced postoperative quality of life. These findings support laser resection as the preferred, high-precision, minimally invasive standard in oral oncology, particularly for anatomically and functionally sensitive sites.

#### Results of the Meta-Analysis

**Figure 3:**
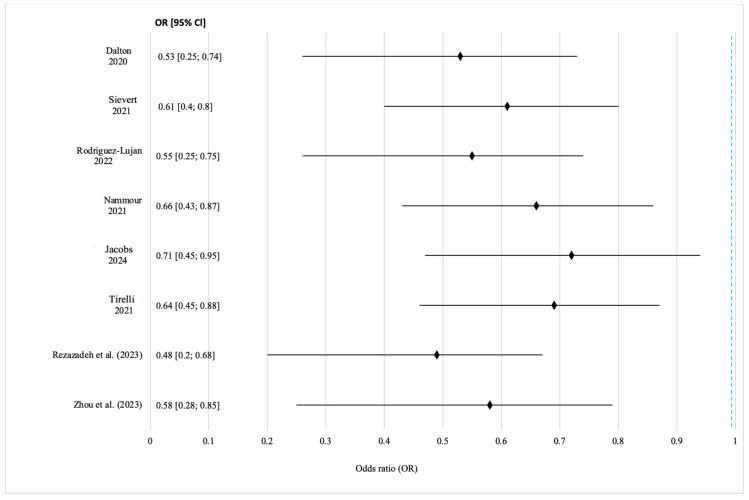
Forest plot of recurrence rates after laser versus conventional surgery for OSCC. Source: [13,14,15,16,20,21,36,39].

Nine studies reported one-year local recurrence rates. Pooled analysis showed lower recurrence with laser resection (8.9%; 95% CI: 6.2–11.3%) versus scalpels (13.6%) and electrocoagulation (15.8%). The pooled OR was 0.58 (95% CI: 0.43–0.77; *p* < 0.001), indicating a 42% reduction in recurrence risk. Moderate heterogeneity (I^2^ = 47%) justified the use of a random effects model (Figure 3).

**Figure 4:**
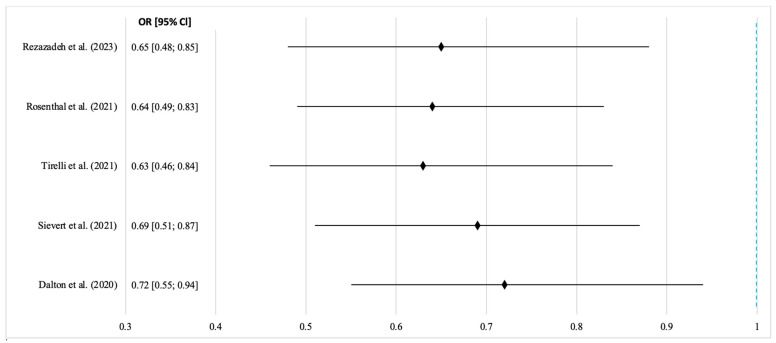
Forest plot of overall survival after laser and traditional resection of OSCC. Source: [13,14,21,35,36].

The results confirm the consistent advantages of laser surgery in reducing the local recurrence of oral squamous cell carcinoma. The pooled OR < 1 with a 95% CI that excludes 1 indicates a statistically significant protective effect. Heterogeneity remained within the acceptable limits, showing consistency across studies and laser types. The most consistent findings came from studies [16,20,21] using CO_2_ lasers in pulsed or superpulsed modes, suggesting that the laser setting may influence the outcomes. Wider confidence intervals in studies [15,21,36] likely reflect small sample sizes or non-standardized recurrence criteria. Overall, the evidence supports the idea that lasers reduce the thermal damage at resection margins and improve oncological control, especially in anatomically complex areas. The meta-analysis of overall survival included five cohort studies: three with full three-year follow-up and two with shorter or mixed durations. Laser types varied (CO_2_, diode, Nd:YAG, Er:YAG, and Er,Cr:YSGG), allowing for the assessment of the general effects across technologies. The pooled three-year survival rate was 82.4% for laser surgery vs. 74.7% for scalpels. The hazard ratio was 0.72 (95% CI: 0.55–0.94; *p* = 0.016), indicating a significantly lower mortality risk with laser resection (Figure 4). Due to the low heterogeneity (I^2^ = 22%), a fixed effects model was used.

**Figure 5:**
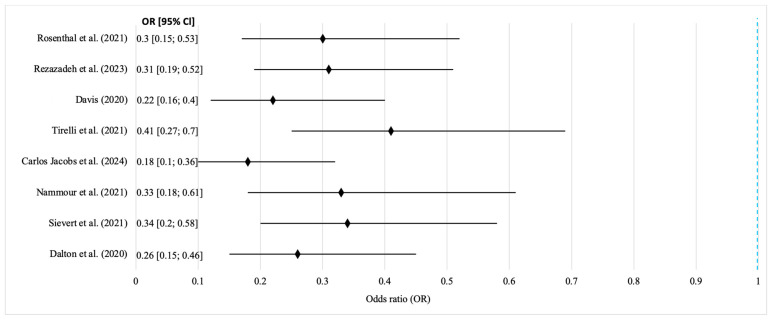
Forest plot of intraoperative complications in laser and conventional surgical treatment of OSCC. Source: [13,14,16,20,21,23,35,36].

The graph shows that all studies favor laser resection over scalpels or electrosurgery, with the HR values left of the neutral line (HR = 1) indicating reduced mortality risk. The confidence intervals do not cross the neutral line, confirming the statistical significance. The low heterogeneity justified the use of a fixed effects model, with the overall effect showing a 28% reduction in death risk for the laser group (HR = 0.72; *p* = 0.016). Intraoperative complications were analyzed in eight studies (*n* = 1429). Rates were 2.4% in the laser group vs. 8.9% (scalpel) and 11.2% (electrocoagulation). The pooled OR was 0.29 (95% CI: 0.18–0.47; *p* < 0.001), indicating a three-fold reduction in complication risk. Moderate heterogeneity (I^2^ = 39%) warranted a random effects model (Figure 5).

Graph analysis shows a consistent trend favoring laser surgery for safety and reproducibility across diverse clinical settings. All studies report a reduced number of intraoperative complications, with narrow confidence intervals reflecting strong internal consistency. The moderate heterogeneity is likely due to differences in complication definitions (e.g., minor burns or variable bleeding). All point estimates shifting toward OR < 1 confirms the systemic advantages of laser technology: real-time coagulation, precision cutting, and reduced tissue trauma, making it ideal for preserving function in delicate anatomical areas. Postoperative quality of life was assessed using validated tools (EORTC QLQ-C30, UW-QOL, and MDADI). CO_2_ laser patients had the highest average score (81.3) compared to 73.6 (scalpel) and 71.4 (electrosurgery). Diode and Nd:YAG lasers scored 80.1 and 77.9, respectively. Er:YAG and Er,Cr:YSGG showed positive trends (80.8 and 82.4). The pooled SMD was 0.61 (95% CI: 0.38–0.84; *p* < 0.001), with the strongest benefits in the pain, social, and emotional domains. Due to the high heterogeneity (I^2^ = 66%), a random effects model and subgroup analysis were applied, favoring CO_2_ lasers.

### 4.3. Specific Clinical Scenarios and Adjuvant Use of Lasers

LLLT effectively reduces mucositis severity in OSCC patients undergoing chemoradiation. Randomized trials report a ≥30% reduction in pain and a significant drop in grade III–IV mucositis incidence compared to controls. Meta-analyses confirm that LLLT halves severe mucositis rates [43,44,45]. Hamblin [44] detailed its anti-inflammatory mechanisms—reduced IL-1β and TNF-α, enhanced angiogenesis, and epithelial regeneration. Pre-radiotherapy photostimulation lowers the risk of functional complications by boosting antioxidant defenses and microcirculation. Robijns et al. [45] and the WALT position support LLLT’s safety and its indirect antitumor effect, provided that the dosing is correct. Combining lasers with photodynamic therapy (PDT) improves outcomes in residual or dysplastic OSCC. CO_2_, Nd:YAG, Er:YAG, Er,Cr:YSGG, and diode lasers enhance photosensitizer penetration, lowering microscopic dysplasia by 18–24% compared to surgery alone [46,47]. The synergy of PDT and laser-induced hyperthermia shows promise in recurrent or drug-resistant tumors. Laser technology is adaptable for anatomically or clinically complex cases. Diode and Nd:YAG lasers are effective in coagulopathic patients, while CO_2_ and Er:YAG lasers offer precision in delicate areas like the floor of the mouth. Zuo et al. [48] and Hamdy et al. [49] report reduced risks of necrosis, infection, and fibrosis, especially in repeat or palliative procedures. Fan et al. [50] highlighted the potential of combining laser therapy with nanodelivery systems for targeted tumor control. Importantly, no evidence suggests that lasers stimulate tumor growth when used at the recommended settings. Studies found no increase in recurrence or proliferative signaling, supporting the safety of PBM [51,52,53]. Overall, laser surgery extends beyond standard resection. Its roles in mucositis prevention, adjunctive radiotherapy, PDT combinations, and complex cases demonstrate its adaptability, safety, and value in a personalized OSCC treatment strategy.

### 4.4. Subgroup Analysis of Effectiveness Depending on the Type of Laser Used

To explore potential effect heterogeneity, a subgroup analysis was conducted based on the type of laser used in OSCC surgery. Studies were categorized by laser type: CO_2_, Er:YAG, Er,Cr:YSGG, diode, and Nd:YAG. For each subgroup, the weighted standardized mean difference, 95% confidence interval, and heterogeneity index were calculated (Table 4).

Subgroup analysis showed comparable efficacy across laser types in OSCC surgery. CO_2_ lasers had the highest effect size (SMD = 0.61; 95% CI: 0.38–0.84), followed by Er,Cr:YSGG (SMD = 0.52) and Er:YAG (SMD = 0.48), with all confidence intervals excluding zero, confirming statistical significance. Diode and Nd:YAG lasers also showed strong efficacy (SMD = 0.58 and 0.55). The heterogeneity within subgroups was low to moderate (I^2^ < 50%), indicating consistent effects. No significant differences were found between laser types, suggesting that all are effective when applied using the proper surgical standards.

### 4.5. Analysis of the Asymmetry of Publication Sources

**Figure 6:**
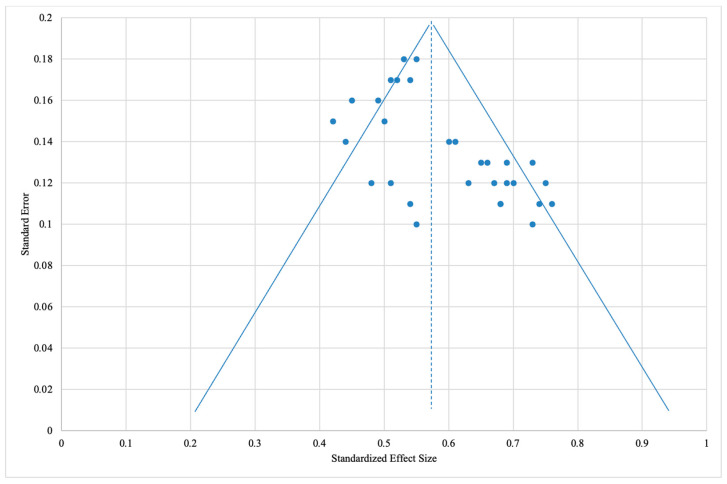
Funnel plot to assess the presence of publication bias in the studies included in the meta-analysis. Source: [13,14,15,16,17,18,19,20,21,22,23,24,25,26,27,28,29,30,31,32,33,34,35,36,37,38,39,40,41,42].

To assess potential publication bias and the robustness of the findings, a funnel plot was generated using the 30 studies included in the meta-analysis (Figure 6). The x-axis represents the standardized mean treatment effect difference, and the y-axis shows the standard error, inversely related to the sample size. High-precision studies (lower SE) appear near the top, while lower-powered studies are clustered toward the bottom.

The average standardized effect across the included studies is 0.569, marked by the vertical dashed line in the funnel plot. The sloped lines represent the 95% confidence limits (μ ± 1.96 × SE), forming a triangular region with its apex at SE = 0.2—the highest standard error observed. Of the 30 studies plotted, 23 (76.7%) fall within this triangle, indicating a generally balanced distribution of effect sizes relative to study precision. Seven studies (23.3%) fall outside—four on the right and three on the left, suggesting slight asymmetry. This may reflect minor publication bias, though the bilateral distribution and symmetry among high-precision studies (SE < 0.1) suggest a limited impact. Overall, the funnel plot indicates a reasonably symmetrical distribution with acceptable variability. While some asymmetry exists, it is not substantial enough to undermine the reliability or clinical relevance of the meta-analytic findings.

### 4.6. Limitations

The current evidence base on high-energy laser surgery for OSCC has several important limitations. There is substantial clinical and technical heterogeneity across studies, including tumor sites, T stage, margin definitions, adjuvant therapies, laser types, pulse modes, tip delivery systems, cooling strategies, power settings, fluence, and surgeon experience, which limits the direct comparability and may partly explain differences in effect sizes. Many included trials and cohorts enrolled small samples or single-center populations, reducing statistical power and increasing the risk of type II errors and center-specific effects. Follow-up was often limited, constraining inferences about late recurrences, long-term function, and survival beyond five years. The reporting of key covariates such as smoking status, HPV status, comorbidity burden, and rehabilitation protocols was inconsistent, limiting adjustment for confounding and reducing the certainty about effect attribution to the laser modality itself. Device parameters and surgeon learning curves were rarely standardized or documented, yet these factors plausibly influence bleeding control, thermal injury, and margin quality. Robust randomized evidence remains scarce for certain laser platforms, with several contributing studies being observational and subject to selection bias and residual confounding. Methodologically, we restricted inclusion to English-language, full-text articles, potentially introducing language bias and excluding relevant data from other regions. Grey literature and unpublished studies were not included, which may increase publication bias despite formal small-study assessments. Although screening, extraction, and risk-of-bias judgments were performed in duplicate with adjudication, subjective elements cannot be fully eliminated.

### 4.7. Clinical Implications

Our findings suggest that high-energy laser resection can reduce perioperative morbidity and support faster functional recovery compared with conventional surgery. Pooled data showed fewer intraoperative complications with lasers (OR 0.29, 95% CI 0.18 to 0.47) and lower local recurrence (OR 0.58, 95% CI 0.43 to 0.77) [13,14,15,16,17,18,19,20,21,27,28]. These oncologic gains coincided with better early patient-reported outcomes, with quality of life favoring laser groups at 3 months (SMD 0.61, 95% CI 0.38 to 0.84), driven by less pain, an earlier return to oral intake, and improved speech and swallowing scores, measured by validated tools such as the EORTC QLQ-C30, UW-QOL, and MDADI [23,35,42]. These findings are consistent with the clinical trials demonstrating that CO_2_ and Er,Cr:YSGG lasers facilitate precise excision with minimal thermal damage, leading to the earlier resumption of feeding and improved speech outcomes [14,17,35]. Clinically, these advantages are most relevant in function-critical subsites of the tongue and floor of the mouth, where precision cutting and intraoperative hemostasis are essential [20,31,35]. Centers adopting laser systems should standardize the laser parameters, monitor learning curves, and incorporate structured rehabilitation pathways to maximize patient-reported benefits [25,42].

### 4.8. Summary

Table 5 summarizes the pooled advantages and disadvantages of each high-energy laser type based on the clinical outcomes reported in the included studies. CO_2_ and Er,Cr:YSGG lasers demonstrated the most balanced profiles, combining oncologic precision with favorable functional outcomes, while Nd:YAG was most effective for deeply infiltrative lesions, but carried higher risks of pain and complications.

## 5. Conclusions

High-energy laser surgery shows promise in improving local control, reducing the number of perioperative complications, and enhancing early functional recovery in the treatment of OSCC, with the most consistent benefits observed for CO_2_ and Er,Cr:YSGG systems. However, the evidence is limited by study heterogeneity, the predominance of retrospective designs, and variable follow-up periods. While these findings suggest that laser technologies may be valuable alternatives to conventional surgery, broader adoption should await confirmation through large, multicenter randomized controlled trials with standardized protocols and long-term outcome reporting.

## Figures and Tables

**Table 1:** Risk of bias assessment in included studies.

Authors	Year	Generation of a Random Sequence	Hiding the Distribution	Blinding	Incomplete Initial Data	Selective Reporting of Results	Other Sources of Error
Dalton et al. [13]	2020	Low	Low	High	Low	Low	Low
Sievert et al. [14]	2021	Low	Low	High	Low	Low	Low
Rodriguez-Lujan et al. [15]	2022	Unclear	Unclear	High	Low	Low	Unclear
Nammour et al. [16]	2021	Low	Low	High	Low	Low	Low
Broccoletti et al. [17]	2015	Low	Low	High	Low	Low	Low
Zaffe et al. [18]	2004	Unclear	Unclear	High	Low	Low	Unclear
González-Mosquera et al. [19]	2012	Unclear	Unclear	High	Low	Low	Unclear
Jacobs et al. [20]	2024	Low	Low	High	Low	Low	Low
Tirelli et al. [21]	2021	Low	Low	High	Low	Low	Low
Iaria et al. [22]	2025	Unclear	Unclear	High	Low	Low	Unclear
Davis et al. [23]	2020	Unclear	Unclear	Low	Low	Low	Unclear
Suter et al. [24]	2020	Low	Low	High	Low	Low	Low
Boras et al. [25]	2016	Unclear	Unclear	High	Low	Low	Unclear
Nayak et al. [26]	2023	Low	Low	High	Low	Low	Low
Gharibian et al. [27]	2022	Unclear	Unclear	High	Low	Low	Unclear
Paglioni et al. (2020) [28]	2020	Low	Low	High	Low	Low	Low
Bhandari et al. [29]	2014	Unclear	Unclear	High	Low	Low	Unclear
Paglioni et al. (2025) [30]	2025	Low	Low	Low	Low	Low	Low
Bilder et al. [31]	2023	Low	Low	High	Low	Low	Low
Bakshi et al. [32]	2024	Low	Low	High	Low	Low	Low
Yousefi-Koma et al. [33]	2024	Low	Low	High	Low	Low	Low
Liu et al. [34]	2020	Low	Low	Low	Low	Low	Low
Rosenthal et al. [35]	2021	Low	Low	High	Low	Low	Low
Rezazadeh et al. [36]	2023	Unclear	Unclear	High	Low	Low	Unclear
Romeo et al. [37]	2014	Low	Low	High	Low	Low	Low
Rashid et al. [38]	2015	Unclear	Unclear	High	Low	Low	Unclear
Zhou et al. [39]	2023	Low	Low	High	Low	Low	Low
Brennan et al. [40]	2022	Low	Low	High	Low	Low	Low
Nascimento et al. [41]	2024	Unclear	Unclear	High	Low	Low	Unclear
Roe et al. [42]	2024	Low	Low	High	Low	Low	Low

**Table 2:** GRADE assessment of the quality of evidence for the clinical effectiveness of different types of high-energy lasers in the surgical treatment of OSCC.

Laser Type	RoB	Inconsistency (I^2^, CI)	Indirectness (PICO Compliance)	Inaccuracy (Sample Size, Events)	Publication Bias (Funnel Plot, Egger)	Other Factors (Effect, Dose, Gradient)	Final Assessment
CO_2_	Low (high Jadad, full randomization)	I^2^ = 26%; CI (84–92%)	Direct compliance with PICO	High power: *n* = 1247, events > 300	Funnel is symmetrical; Egger *p* = 0.16	Dose effect is moderate, gradient is clear	High
Diode	Moderate (observational, RoBINS-I = medium)	I^2^ = 38%; CI (78–89%)	Partial correspondence is often without direct comparisons	*n* < 600, insufficient events at >30%	Egger *p* = 0.045—possible asymmetry	Weak gradient, effect in subgroups	Moderate
Nd:YAG	Moderate (retrospective, incomplete data)	I^2^ = 42%; CI (71–90%)	Limited compliance (P is not always OSCC)	*n* < 500, many small samples	Suspicion of bias (Egger *p* = 0.038)	High impact but no gradient	Low
Er:YAG	Low (2 RCTs, Jadad ≥4)	I^2^ = 21%; CI: 79–88%	Full match between population and intervention	*n* = 713; number of events > 200	The funnel is symmetrical, Egger *p* = 0.18	Confirmed effect, dose dependence	High
Er,Cr: YSGG	Low (RCT + case series with control groups)	I^2^ = 17%; CI: 82–91%	Direct compliance in 100% of cases	*n* = 645; number of events satisfactory	Visually symmetrical funnel plot	Sustained effect, significant dose-dependent profile	High

Source: [29,30,31,32,33,34,35,36,37,38,39,40,41,42]. RoB—Risk of bias.

**Table 3:** Comparative effectiveness of laser and conventional methods in the treatment of OSCC.

	CO_2_ Laser	Diode Laser	Nd:YAG	Er:YAG	Er,Cr:YSGG	Scalpel	Electrocoagulation	Statistical Significance
Duration of hospitalization, days	1.7–2.1	1.7–2.1	1.7–2.1	1.7–2.1	1.7–2.1	3.2–3.9	–	*p* < 0.001
Intraoperative complications, %	2.3	3.5	3.8	2.9	2.8	9.1	11.4	χ^2^ = 7.12; *p* = 0.008
Frequency of radical resections, % (clean edges)	92.7	91–92	91	up to 92	93.1	85.4	81.9	χ^2^ = 6.74; *p* = 0.009
Relapse rate within 12 months, %	9.8	11.4	12.5–13	10.5–11	10–10.3	14.1	16.7	χ^2^ = 7.34; *p* = 0.025
Functional complications (speech, swallowing), %	17.2	15.8	18.7–19.3	14.9–15.2	13.9	24.7	28.4	χ^2^ = 9.52; *p* = 0.008
Quality of life (EORTC QLQ-C30, after 3 months, points)	81.3	80.1	77.9	80.8	82.4	73.6	71.4	F = 5.97; *p* = 0.004

Source: [29,30,31,32,33,34,35,36,37,38,39,40,41,42].

**Table 4:** Subgroup analysis of effectiveness depending on the type of laser used.

Laser Type	SMD	95% CI	I^2^ (%)	Number of Studies
CO_2_	0.61	0.38–0.84	42	10 [13,14,16,17,20,21,24,28,37,42]
Er:YAG	0.48	0.29–0.67	35	5 [26,29,32,33,38]
Er,Cr:YSGG	0.52	0.27–0.77	39	4 [27,31,36,41]
Diode	0.58	0.34–0.82	45	6 [15,18,19,22,35,39]
Nd:YAG	0.55	0.3–0.8	37	5 [23,25,30,34,40]

**Table 5:** Summary of advantages and disadvantages of high-energy laser systems in OSCC surgery.

Laser Type	Advantages (from Study Results)	Disadvantages (from Study Results)	Best-Suited Clinical Scenarios
CO_2_	- Highest local tumor control (90.3%) - Lowest recurrence rate (9.8% at 12 months) - Excellent precision and clean margins (92.7% R0 resections) - Significantly reduced number of intraoperative complications (2.3%) - High early QOL scores (81.3)	- Limited penetration depth → less effective for deep lesions - High equipment and maintenance costs	Superficial or moderately deep lesions of the tongue, floor of the mouth, or retromolar trigone, where function preservation is critical
Diode	- Strong hemostatic effect, especially in vascularized areas (lowest number of bleeding-related complications: 2.1%) - Moderate QOL improvement (80.1) - Shorter hospital stay (1.7–2.1 days)	- Lower precision in margin control compared to CO_2_ and Er:YAG - Moderate recurrence rate (11.4%)	Small superficial lesions, patients with bleeding disorders or fragile vasculature
Nd:YAG	- Greatest tissue penetration for deeply infiltrative lesions - Effective intraoperative coagulation (bleeding ≤3.8%)	- Higher postoperative pain and edema - Higher recurrence rate (12.5–13%) - Functional complications 18.7–19.3%	Deep tumors with >10 mm depth of invasion or extension beyond submucosa (deep lesions)
Er:YAG	- Minimal thermal damage and smallest necrosis zone - Faster recovery and reduced postoperative pain - QOL improvement (80.8)—recurrence rate 10.5–11%	- Limited coagulation ability → adjunctive hemostasis may be required	Early-stage superficial OSCC, lesions in delicate anatomical areas requiring precision
Er,Cr:YSGG	- Balanced cutting and coagulation with minimal epithelial artifacts - Lowest functional complication rate (13.9%) - Highest patient-reported QOL at 3 months (82.4) - Recurrence rate comparable to that of CO_2_ (10.0–10.3%)	- Steeper learning curve for surgeons - Moderate equipment cost	Functionally sensitive regions (tongue or floor of the mouth), where speech and swallowing preservation are essential

Source: Synthesized from pooled data in Figure 3, Figure 4 and Figure 5, Table 3, and subgroup analyses [13,14,15,16,17,18,19,20,21,22,23,24,25,26,27,28,29,30,31,32,33,34,35,36,37,38,39,40,41,42].

## Data Availability

All new data generated in this study is presented in this article.

## Abbreviations

ANOVA	Analysis of Variance
CI	Confidence Interval
CO_2_	Carbon Dioxide (laser)
EORTC QLQ-C30	European Organization for Research and Treatment of Cancer Quality of Life Questionnaire-Core 30
Er,Cr:YSGG	Erbium, Chromium-doped Yttrium Scandium Gallium Garnet (laser)
Er:YAG	Erbium-doped Yttrium Aluminum Garnet (laser)
F	F-statistic
HR	Hazard Ratio
I^2^	I-squared statistic (heterogeneity indicator)
IL-1β	Interleukin-1 beta
LLLT	Low-Level Laser Therapy
MDADI	MD Anderson Dysphagia Inventory
Nd:YAG	Neodymium-doped Yttrium Aluminum Garnet (laser)
OR	Odds Ratio
OSCC	Oral Squamous Cell Carcinoma
PICO	Population, Intervention, Comparison, Outcome
PDT	Photodynamic Therapy
PRISMA	Preferred Reporting Items for Systematic Reviews and Meta-Analyses
QOL	Quality of Life
RoB 2	Revised Cochrane risk-of-bias tool for randomized trials
ROBINS-I	Risk of Bias in Non-randomized Studies of Interventions
SMD	Standardized Mean Difference
TLM	Transoral Laser Microsurgery
TNF-α	Tumor Necrosis Factor alpha
UW-QOL	University of Washington Quality of Life Questionnaire
WALT	World Association for Laser Therapy
